# DNA methylation in poultry: a review

**DOI:** 10.1186/s40104-023-00939-9

**Published:** 2023-11-05

**Authors:** Xing Ju, Zhijun Wang, Danfeng Cai, Semiu Folaniyi Bello, Qinghua Nie

**Affiliations:** 1 State Key Laboratory for Conservation and Utilization of Subtropical Agro-Bioresources, Lingnan Guangdong Laboratory of Agriculture, College of Animal Science, South China Agricultural University, Guangzhou, Guangdong 510642 China; 2 Guangdong Provincial Key Lab of Agro-Animal Genomics and Molecular Breeding and Key Lab of Chicken Genetics, Breeding and Reproduction, Ministry of Agriculture, Guangzhou, Guangdong 510642 China; 3 College of Animal Science and Technology, Zhejiang Agriculture and Forestry University, 666 Wusu Road, Lin’an, 311300 China

**Keywords:** CpG islands, Differentially methylated genes, Differentially methylated regions, DNA methylation, Poultry

## Abstract

As an important epigenetic modification, DNA methylation is involved in many biological processes such as animal cell differentiation, embryonic development, genomic imprinting and sex chromosome inactivation. As DNA methylation sequencing becomes more sophisticated, it becomes possible to use it to solve more zoological problems. This paper reviews the characteristics of DNA methylation, with emphasis on the research and application of DNA methylation in poultry.

## Introduction

The maturation of molecular genetic marker technology makes the quantitative trait map of livestock and poultry robust systematic and perfect, and provides a new means for livestock and poultry improvement. DNA methylation is a widely used epigenetic modification [1–6]. DNA methylation causes the activity of certain genes to be turned off, while demethylation induces gene reactivation and expression [7, 8]. Under the catalytic action of DNA methyltransferase, the two nucleotides of CG in DNA are selectively added methyl groups to form 5mC, which mostly appears in the 5'-CG-3' gene sequence [9, 10]. Most vertebrate genome DNA contains a small amount of methylated C, which is mainly distributed in the non-coding region at the 5' end of the gene and exists in clusters [11–14].

DNA methylation has been widely utilized in poultry species [15–21]. For example, in the DNA methylation map of broilers, there are more hypomethylated regions in the genome, and CGIs (CpG islands) has the largest distribution in the gene promoter region, indicating that the hypomethylation of CGIs methylation and muscle developing-related genes is involved in the rapid muscle development of broilers. Meanwhile, MyH1-AS, a lncRNA (long non-coding RNA) present in the DMRs (differential methylation regions) is involved in regulating the development of chicken embryonic muscle in chicken [22]. In the DNA methylation study of skeletal muscle satellite cells, the Wnt signaling pathway was significantly enriched in Kyoto Encyclopedia of Genes and Genomes database and Gene Ontology, and the methylation status of promoter region affected the expression levels of *Wnt5a*, *Wnt9a* and *TGFβ1* genes, suggesting that the methylation status of Wnt and TGFβ signals is a key regulatory factor during skeletal muscle development [23]. These markers are of great significance for understanding the molecular regulation mechanism and genetic expression mechanism of important economic traits of poultry and promoting poultry genetic breeding.

**Fig. 1:**
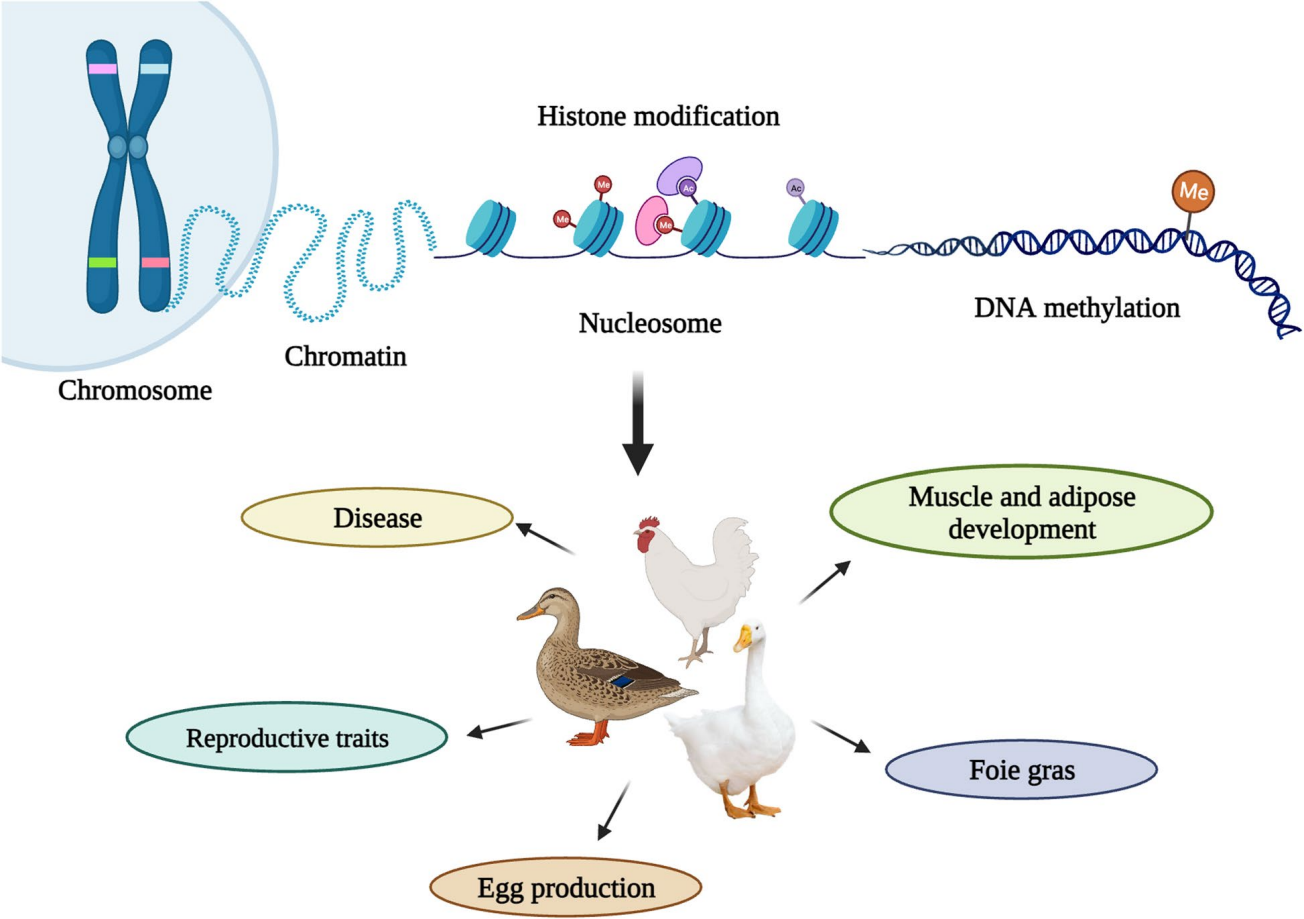
Overview of epigenetic mechanisms in poultry

With the continuous development of sequencing technology, DNA methylation epigenetic research has been widely studied in the fields of biology, medicine, agriculture and forestry. Numerous studies at the genome level have been increasing the understanding of the genetic mechanism of important economic traits in livestock and poultry, reducing the occurrence of diseases and genetic defects in breeding, and making important contributions to improving the production efficiency, product quality and economic benefits of poultry (Fig. 1). Through the review of the relevant research content of poultry DNA methylation, we further understand the role of DNA methylation in poultry production, which may provide a certain theoretical basis.

## The application of DNA methylation in poultry reproductive traits

*Gallus gallus* (domestic chickens) are the world’s most important commercial source of meat, however, there has been less research into chicken epigenetics [15, 24–26]. One of the important problems in the protection of chicken genetic resources is the suppression of chicken inbreeding [1, 27, 28].

**Fig. 2:**
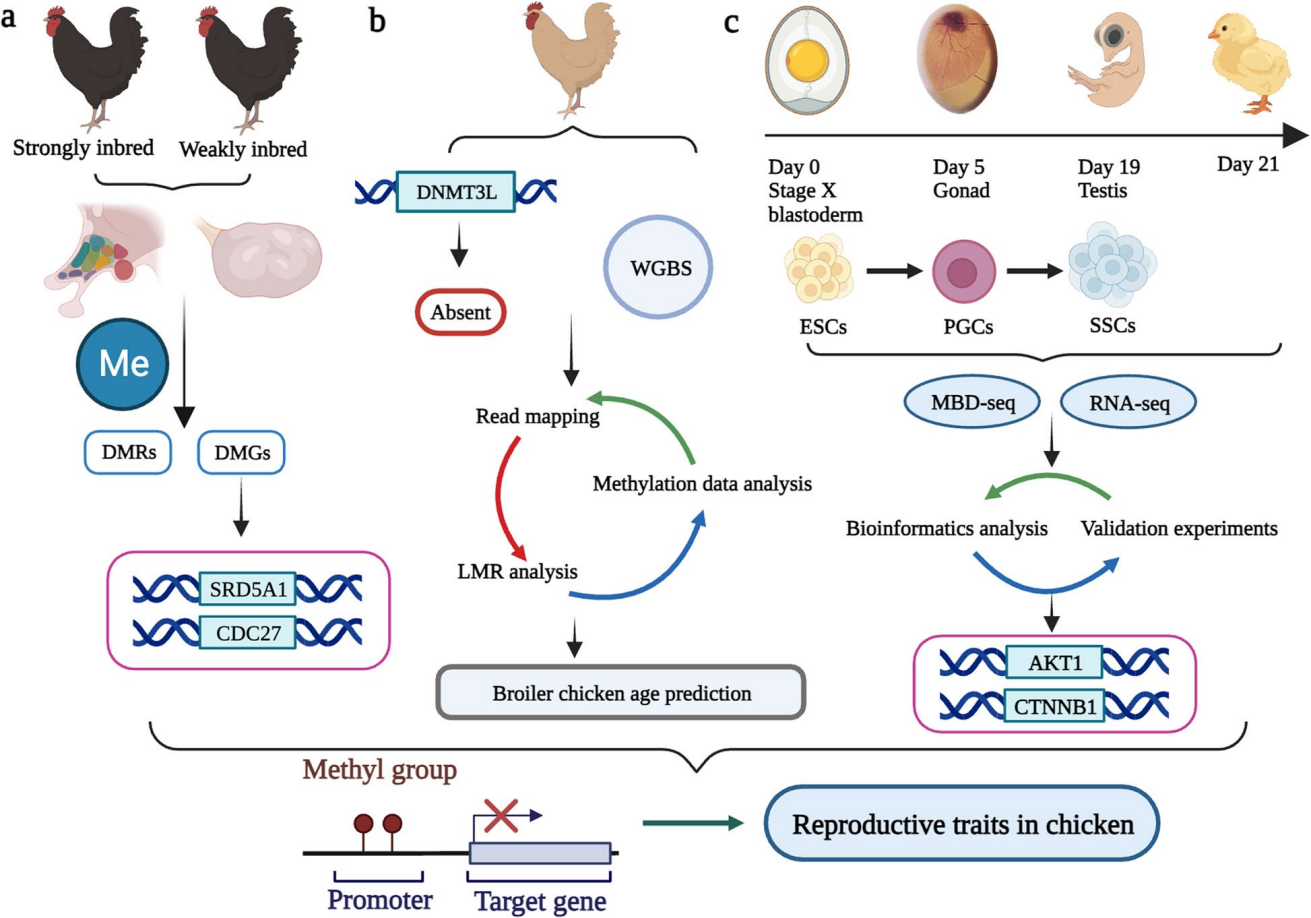
Application of DNA methylation to reproductive traits in poultry. **a** Through DNA methylation analysis of the hypothalamus and ovary of strongly inbred chickens, the marker genes *SRD5A1* and *CDC27*, which may be the inhibition of inbreeding of Langshan chickens, were found in the DMRs region. **b** Although the overall distribution of DNA methylation was similar to that of mammals, sperm DNA showed hypomethylation, which was related to the deletion of *DNMT3L* cofactor in the chicken genome. And a DNA methylation clock based on LMR was established for broiler age prediction. **c** A comprehensive genome-wide DNA methylation landscape in chicken germ cells was reported. And by revealing the DNA methylation patterns of individual genes, it was found that some genes precisely regulated by DNA methylation were associated with cancer and viral infections, such as *AKT1* and *CTNNB1*

At present, common epigenetic DNA methylation research sequencing methods include MeDIP-Seq (methylated DNA immunocoprecipitation sequencing), MBD-Seq (methylated DNA binding domain sequencing) and WGBS (whole genome DNA methylation sequencing) [29, 30]. Although, few studies have sequenced chicken reproductive related tissues by these sequencing methods. The effects of DNA methylation on chicken reproductive performance were investigated and some key molecular markers were found. WGBS was performed and 5,948 and 4,593 DMRs were identified in the hypothalamus and ovary of strong and weak inbred Langshan chickens, respectively. A large number of DMGs (DMR related genes) were enriched in reproductive related pathways. A study combined with WGBS and transcriptome data and concluded that two DMRs in the *SRD5A1* and *CDC27* genes may be biomarkers of inbred reproductive inhibition in Langshan chickens [31] (Fig. 2a). Nevertheless, analysis of the chicken DNA methylation mechanism and DNA methylation landscape revealed that the overall distribution of DNA methylation was similar to that of mammals and sperm DNA showed hypomethylation, which was associated with the deletion of *DNMT3L* cofactors in the chicken genome. In addition, the study provided its dynamic regulation at transcription factor binding sites, and this information was applied to construct chicken DNA methylation clocks that can accurately predict the age of broilers [32] (Fig. 2b). An important new approach in human medicine and stem cell biology is the production of germ cells in vitro [33–35]. By revealing the DNA methylation patterns of individual genes, it was found that certain genes such as *AKT1* and *CTNNB1 *precisely regulated by DNA methylation were associated with cancer and viral infections. Chicken-specific markers used to identify male germ cells were also revealed. Importantly, this study explored the integrated epigenetic mechanisms of male germ cell differentiation [36] (Fig. 2c).

DNA methylation is the link between genes and phenotypes [17–19, 37, 38], and has been widely used to identify environmental influences on poultry growth [16, 39–41]. DNA methylation has also been studied in ducks, not just chickens.

The differences of DNA methylation between breeding and protective populations of Shaoxing ducks were investigated by genome-wide DNA methylation detection. Thirty-five differentially methylated genes were identified and these genes were closely related to production performance. In addition, *ATP2B1* and *ATP2B2* genes related to eggshell quality were also identified as differentially methylated, which could be used as molecular markers to improve eggshell quality in the future [21]. Incubation temperature also has long-term effects on bird embryo development and its effect on DNA methylation was investigated by increasing the incubation temperature from 37.8 to 38.8 ºC at ED (embryonic stage) 1–10, ED10–20 and ED20–27. The results indicated that *Methyl-CpG binding domain proteins* and *DNA (cytosine 5)-methyltransferases* may be involved in the thermoepigenetic regulation of early embryonic development in ducks [20].

## The mechanism of DNA methylation during muscle development in poultry

**Fig. 3:**
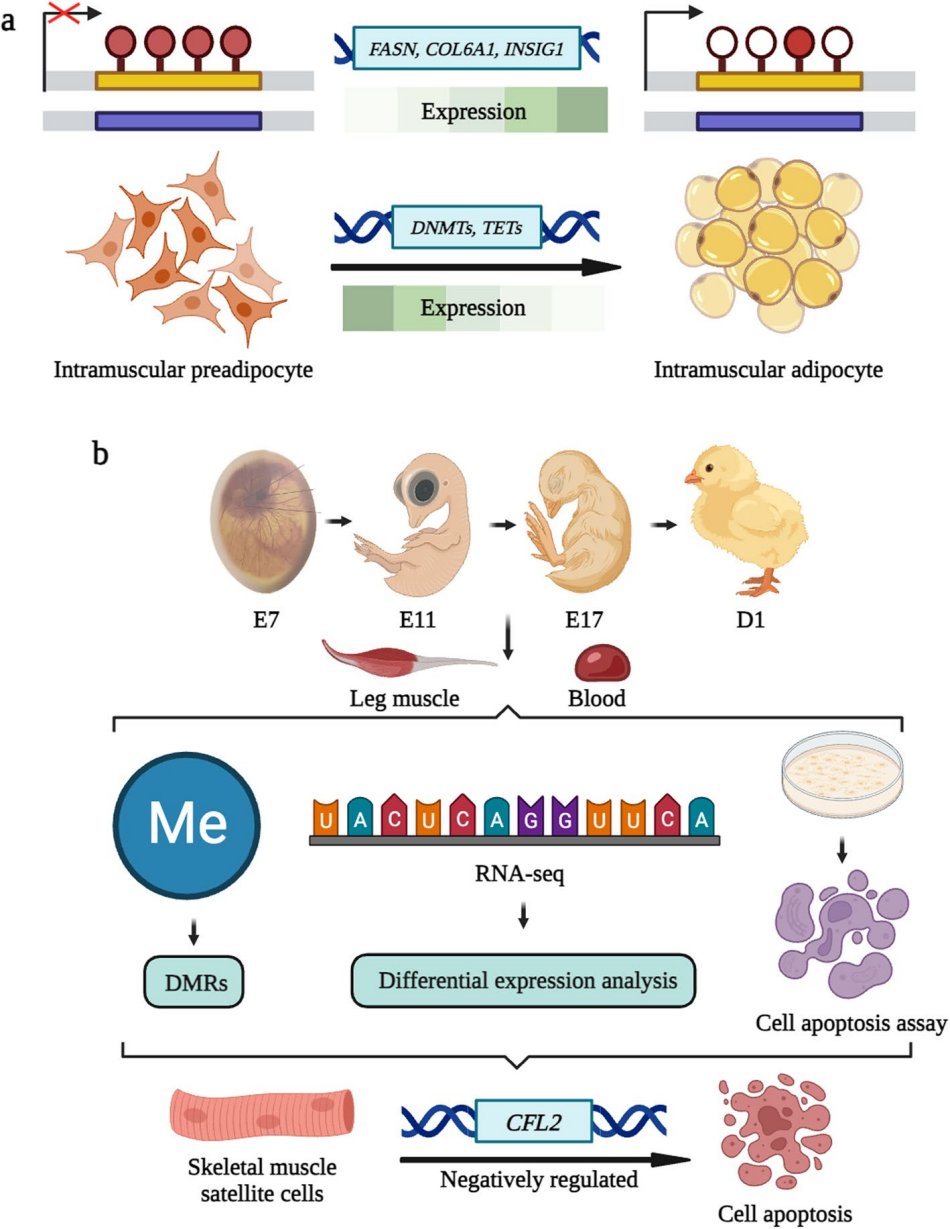
Mechanism of DNA methylation during muscle and adipose development in poultry. **a** DNA methylation regulates intramuscular fat formation by regulating genes such as collagen, type VI and alpha 1 (*COL6A1*), thus affecting IMF deposition. **b** In vitro experiments showed that *CFL2* negatively regulated the proliferation of chicken skeletal muscle satellite cells and induced cell apoptosis

Intramuscular fat (IMF) is an indispensable factor affecting meat quality, which is regulated by nutrition, environment and genetics [42–45]. DNA methylation plays a crucial role in early muscle development [46]. By establishing an intramuscular adipocyte differentiation model, it has been reported that DNA methylation affects IMF deposition by regulating genes such as *COL6A1*, which regulate the formation of intramuscular adipocytes [47] (Fig. 3a). In the early development of muscles, DNA methylation is a significant factor that cannot be ignored [22, 48, 49]. Different studies had found that DMGs were significantly related to actin filament depolymerization, skeletal muscle satellite cell proliferation and muscle organ development while *CFL2* negatively regulated the proliferation of chicken skeletal muscle satellite cells and induced cell apoptosis [50] (Fig. 3b).

## The role of DNA methylation in the development of disease in poultry

**Fig. 4:**
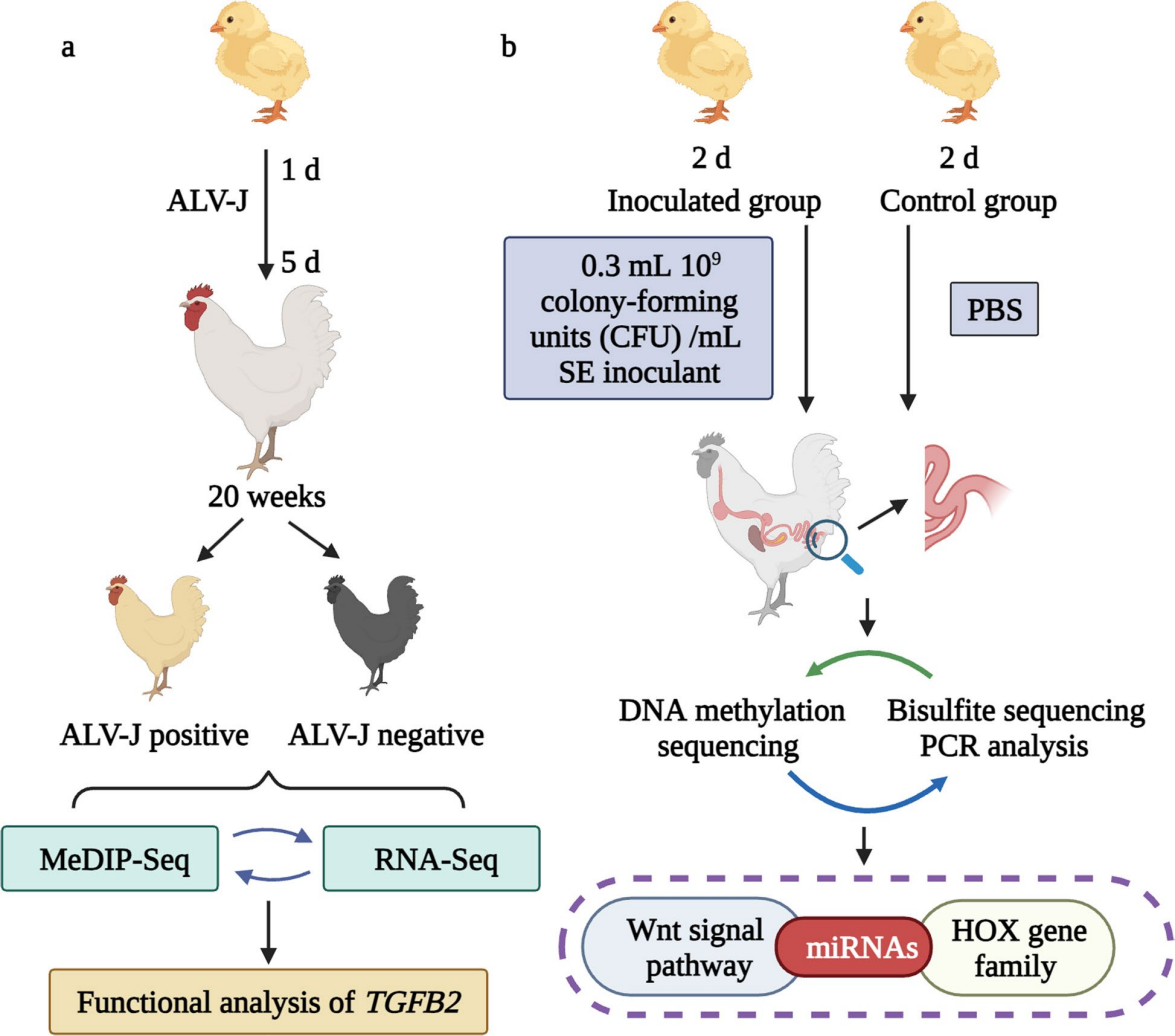
The role of DNA methylation in the development of disease in poultry. **a** MeDIP-seq analysis was used to identify DMRs and RNA-seq analysis was used to identify DEGs in ALV-J positive and negative chicken samples, suggested that *TGFB2* may be an indicator for identifying ALV-J infection. **b** SE inoculation can promote DNA methylation in chicken cecum and cause methylation changes of immune and metabolism-related genes. Wnt signaling pathways, miRNAs and *HOX* gene families may play key roles in the regulation of SE methylation in chicken inoculation

Avian leukosis virus subgroup J (ALV-J) and *Salmonella enterica* serovar Enteritidis (SE) can cause serious economic losses in the poultry industry by affecting poultry production, and pose a serious threat to public health [51, 52]. A growing number of diseases have been shown to be associated with alterations in DNA methylation [53–56]. Genome-wide gene expression and DNA methylation profiles of ALV-J positive and negative chicken samples were generated and provided by MeDIP-seq and RNA-seq (RNA sequencing) studies. Six candidate genes were screened by integration analysis to identify ALV-J negative chickens with differences in methylation of promoter region [52] (Fig. 4a). The whole genome DNA methylation profile of chicken SE reaction was analyzed to reveal the regulatory mechanism of methylation in chicken SE reaction. SE inoculation can promote DNA methylation in chicken cecum and cause methylation changes of genes related to immunity and metabolism. Wnt signaling pathways including miRNAs and HOX gene families may play key roles in the regulation of SE methylation in chicken inoculation [51] (Fig. 4b).

 Cluster of differentiation 8 (*CD8*) acts as a co-receptor of T cell receptors, presenting peptides on the cell surface [57, 58]. *CD8A* transcription is controlled by several *cis*-and *trans*-acting elements and DNA methylation. Xu et al. [59] studied the epigenetic transcriptional regulation mechanism of *CD8A* expression, such as DNA methylation, and found that the *CD8A* mRNA level was negatively releated with the overall methylation status of *CD8A* CpG in ducks thereby suggesting that the hypermethylation of *CD8A* may be related to the hypermethylation of *CD8A* and *DHV-1* infection in ducks.

 Ferritin heavy polypeptide 1 (*FTH1*) expression is regulated by a variety of pathogens, but its regulatory mechanisms remain unclear [60–64]. A duck hepatitis virus (DHV-1) infection model was constructed to detect *FTH1* (*duFTH1*) mRNA expression in ducks infected with DHV-1. The DNA methylation level of *duFTH1* promoter region was detected by BSP (bisulfite sequencing), and the region was found to be hypomethylated. The *duFTH1* promoter region was also found to contain a mutation affecting the activity of the region, which altered the binding site of the transcription factor *NRF1*. The binding of *FTH1* promoter and *NRF1* was confirmed by further analysis. This study provided molecular insights into the influence of *duFTH1* expression on DHV-1 challenges through promoter polymorphism rather than DNA methylation [65].

## How does DNA methylation affect egg production in poultry

Egg production is an important economic trait of poultry [66]. It is of great importance for breeders to understand the key genes that influence egg production.

It has been reported that the effects of VC (vitamin C) feeding on the performance, immune status and expression of DNA methylation-related genes of broilers at embryo age 11 (E11). The results showed that IOF (in ovo feeding) of VC at E11 improved the performance and immune status of broilers after hatching, and increased the antioxidant capacity of broilers to a certain extent. The expression of enzyme-related DNA methylation and demethylation suggested that the spleen DNA methylation level may be increased in the VC group, but whether the expression fluctuation of anti-inflammatory and pro-inflammatory cytokines is related to the changes of DNA methylation remains to be further studied [67].

The effect of *ZP2* promoter methylation on egg production in Jinghai Yellow chickens has been investigated. By constructing a missing promoter vector of *ZP2* gene, it was predicted that the core region of *ZP2* gene was located between −1,552 and −1,348. The methylation of mC-20 and mC-21 sites in *ZP2* gene promoter region was negatively correlated (*P* < 0.05) with mRNA expression. Both sites are located at the Sp1 transcription factor binding site, and the binding of Sp1 to DNA may be inhibited, thus affecting the transcription of *ZP2* gene and egg production [68].

## Effect of DNA methylation on foie gras

Foie gras is a popular delicacy. It has a lot of unsaturated fatty acids to give it its unique flavor, and it’s loved by consumers all over the world [69–71]. Studies established control and overfeeding group to evaluate the effects of addition of betaine on liver weight and other indicators. The results showed that the expression level of *S14α* mRNA in liver of geese treated with betaine was higher than that of control group and overfed geese. A single allele in this region (between +374 and −8 base pairs relative to the transcription start site) was sequenced with sodium sulfite, containing 33 CpG dinucleotides. And the overfed group expressing higher *S14α* transcripts, the average methylation rate of 33 CpGs sites was 87.9%. In the control group, this contrasted with 69.6% that showed lower expression of the *S14α* gene (*P* < 0.01). However, methylation at the transcriptional start site did not change significantly between betaine treated geese (82.6%) and overfed geese (87.9%). These results suggested that DNA methylation patterns at the transcription start site of *S14α* genes may be independent of the expression of *S14α* transcripts after betaine addition [72].

*C/EBP-β* is one of the key regulatory factors of hepatic lipid metabolism balance [73]. For futher understand the effects of *C/EBP-β* on lipid accumulation in goose liver, few studies had cloned the DNA of *C/EBP-β*. The results showed that betaine did not directly regulate the methylation, but decreased the expression of *C/EBP-β* gene in geese. These data can lay a foundation for further research on the mechanism of *C/EBP-β* regulating fat metabolism in foie gras and the effect of betaine on the molecular level of fat metabolism genes [74].

Dietary methionine restriction affects growth performance and amino acid metabolism. Supplementing the methyl donor with betaine prevents this interference [75, 76]. The effects of dietary methionine and betaine on growth performance, epigenetic mechanism and transcriptome gene expression of methionine-deficient geese were examined. The results showed that dietary betaine and methionine changed the liver DNA methylation of *LOC106032502* and affected the transcriptional regulatory network of geese [77].

## Conclusion

We mainly reviewed the effects of DNA methylation on reproductive traits, muscle and adipose development, disease, egg production, etc. And some important DNA methylation markers werer mentioned. DNA methylation plays an important role in poultry. Animal DNA methylation involves many aspects such as growth, development, environment and nutrition. DNA methylation affects the development and differentiation of eukaryotic cells by regulating gene expression [78–80]. Numerous studies have utilized DNA methylation as biomarkers for disease recognition and diagnosis, animal growth trait markers, ketone body trait markers, etc. (Table 1).

**Table 1:** Major markers involved in DNA methylation in poultry

Traits	Gene	Description	Methods	Species	Chr	Pathway	Reference
Production	*SRD5A1*	Steroid-5-alpha-reductase, alpha polypeptide 1	WGBS	Langshan chicken	2	Steroid hormone biosynthesis	Han et al. [31]
	*CDC27*	Cell division cycle protein 27 homolog			27	Progesterone-mediated oocyte matur-ation	
	*WFIKKN1*	WAP, follistatin/kazal, immunoglobulin, kunitz and netrin domain containing 1	MBD-Seq and RNA-Seq	Chicken	14	Negative regulation of transforming growth factor beta receptor signaling pathway	He et al. [36]
	*GAS7*	Growth arrest specific 7			18	Protein binding	
	*TMPRSS9*	Transmembrane protease, serine 9			28	Proteolysis	
	*MDM4*	MDM4, p53 regulator			26	Regulation of cell cycle	
Muscle and adipose development	*FASN*	Fatty acid synthase	WGBS and RNA-Seq	Chicken	18	Fatty acid biosynthetic process	Zhang et al. [47]
	*COL6A1*	Collagen type VI alpha 1 chain			7	Platelet-derived growth factor binding	
	*INSIGI*	Insulin induced gene 2		Chicken	7	Negative regulation of fatty acid biosynthetic process	
	*CFL2*	Cofilin 2	WGBS and RNA-Seq	Chicken	5	Skeletal muscle tissue development	Ran et al. [50]
Disease	*TGFB2*	Transforming growth factor beta 2	RNA-Seq	Chicken	3	Extrinsic apoptotic signaling pathway	Yan et al. [52]
	*HOXA3*	Homeobox A3	WGBS	Chicken	2	Cell population proliferation	Wang et al. [51]
	*HOXD12*	Homeobox D12			7	Regulation of DNA-templated transcriptio	
	*CD8A*	Cluster of differentiation 8	RT-qPCR	Duck	4	-	Xu et al. [59]
	*FTH1*	Ferritin heavy polypeptide 1	WGBS	Duck	5	Cellular iron ion homeostasis	Xu et al. [65]
Egg production	*ZP2*	Zona pellucida 2	qRT-PCR	Jinghai yellow chickens	14	Structural constituent of egg coat	Zhang et al. [68]
Foie gras	*S14α*	Thyroid hormone-responsive Spot14	RT-PCR	Landes goose	Unknow	Unknow	Su et al. [72]
	*C/EBPβ*	CCAAT/enhancer-binding protein β	Bisul fite sequencing PCR	Landes goose	Unknow	Unknow	Yu et al. [74]
	*LOC106032502*	Pantetheinase-like [Anas Platyrhynchos]	RNA-Seq	Geese	Unknow	Unknow	Yang et al. [77]
	*HDAC7*	Histone deacetylase 7			Unknow	Unknow	

However, from the existing studies, there were few studies on DNA methylation related to ducks and geese, while there were more studies on chickens. And current research still faces many challenges, such as obtaining samples of certain diseases and animal tissues due to dynamic nature and relative instability of DNA methylation. More so, it is difficult to establish a stable reference model of DNA methylation. We are of the opinion that with the establishment of DNA whole genome methylation bioinformation database and the development of DNA methylation detection technology, the research on poultry DNA methylation will be more and more comprehensive, more DNA methylation markers will be discovered and applied to practical production, and these problems will be gradually solved.

## Data Availability

Not applicable.

## Abbreviations

ALV: Avian leukosis virus
CD8: Cluster of differentiation 8
CGIs: CpG Islands
DMGs: Differentially methylated genes
DMRs: Differentially methylated regions
ED: Embryonic stage
FTH1: Ferritin heavy polypeptide 1
IMF: Intramuscular fat
IOF: In ovo feeding
MBD-Seq: Methylated DNA binding domain sequencing
MeDIP-Seq: Methylated DNA immunocoprecipitation sequencing
RNA-seq: RNA sequencing
SE: *S**almonella* *enterica* serovar Enteritidis
VC: Vitamin C
WGBS: Whole genome DNA methylation sequencing
